# A Quality Control Procedure for Single Fiducial Tracking of Lung Lesions

**DOI:** 10.7759/cureus.83935

**Published:** 2025-05-11

**Authors:** Martina Descovich, Evan Lau, Katelyn Hasse, Alexander Gottschalk

**Affiliations:** 1 Department of Radiation Oncology, University of California San Francisco, San Francisco, USA

**Keywords:** anatomical alignment, breathing motion, dynamic tracking, fiducial migrations, rotational corrections

## Abstract

Real-time adaptive motion management enables the synchronization of radiation delivery with the patient’s breathing motion, thus reducing target margins and normal tissue exposure. The respiratory model is created by correlating the position of external markers placed on the patient’s chest with the internal target position extracted from planar X-ray images. While a fiducial-less tracking algorithm is available for lung lesions with high radiographic contrast, many patients require the implantation of radio-opaque markers for localization. To limit the incidence of complications from the implantation procedure, a single fiducial marker may be implanted. As fiducials can migrate following implantation, it is important to verify the relationship between the tumor and the fiducial prior to treatment. The goal of this technical report is to provide a quality control (QC) procedure to assess the possibility of fiducial migration in lung patients treated on the robotic radiosurgery platform.

## Introduction

Synchrony® is a motion management solution that enables the CyberKnife® system to adapt radiation delivery to a moving target in real time [1]. It is based on a correlation model between the internal target position, extracted from orthogonal x-ray images, and the position of infrared markers placed on the patient's external surface. Synchrony® allows for margin reductions, compensation for breathing pattern variations, a 100% duty cycle without treatment interruptions, and patient comfort through free breathing. If the lesion is visible on the x-ray images, an image recognition algorithm (Xsight® lung tracking) tracks the target based on density differences between the tumor and its surroundings. Tumors visible on x-ray images are typically larger than 10-15 mm in all dimensions and located in the peripheral region or upper lobes. In a retrospective analysis, Xsight® lung tracking successfully tracked 64% and 81% of lung tumors in 2-view and 1-view configurations, respectively [2]. Evidence shows that lung tracking is a safe and effective treatment option for patients with medically inoperable stage I non-small cell lung cancer [3]. Small lesions and poor radiographic contrast preclude Xsight® lung tracking and necessitate the implantation of radio-opaque fiducial markers for tumor localization. In contemporary lung stereotactic body radiation therapy (SBRT) practices, patients may present with small early-stage lesions due to improved lung nodule detection methods [4]. Three or more fiducials are needed to calculate and compensate for tumor rotations (roll, pitch, and yaw). Fiducial markers provide a robust method for target identification, and accuracy on the order of 3-5 mm has been reported for fiducial-based real-time treatment adaptation [5-7].

All fiducial implantation procedures (percutaneous transthoracic CT-guided insertion, endobronchial ultrasound and electromagnetic navigation bronchoscopy, endovascular delivery of embolization coils) carry some risk of complications, including pneumothorax, pulmonary hemorrhage, air embolism, pulmonary infarction, pleuritic chest pain, and groin hematoma [8]. The risk of complication is related to the needle size and the number of needles inserted per procedure. As a risk mitigation strategy, a single fiducial marker may be implanted in the lesion during the biopsy or as an additional procedure. For example, in re-treatment cases, only one previously implanted fiducial may be available, or additional implantation may be contraindicated due to lesion accessibility, patient comorbidities, or procedural risks. For fiducial stabilization, the simulation CT scan is acquired one week post-implantation. A breath-hold CT allows point-like fiducial identification without motion artifacts. The exhale breath-hold is preferred, as this phase is typically most reproducible and most representative of natural patient breathing. Although a 4D CT scan is not necessary for target contouring and planning, it provides useful information on tumor motion and the target-to-fiducial relationship at the time of simulation.

The accurate relationship between the fiducial and the tumor needs to be verified prior to treatment. While onboard CT provides this validation based on 3D anatomical information, the soft-tissue contrast of planar x-ray images is not sufficient. In the case of multiple fiducials, the difference in inter-fiducial distance between the x-ray images and the digitally reconstructed radiograph (DRR) (rigid body error) provides information on the stability of the fiducial configuration. In the case of a single fiducial, rigid body error and rotational corrections are not calculated, and there is a risk of fiducial migration.

The American Association of Physicists in Medicine (AAPM) Task Group report TG-135B [9] recommends using a spine setup plan to adjust rotations for fiducial cases, similar to the Xsight® lung tracking workflow. In this report, we propose a workflow to evaluate potential fiducial migration based on the spine setup.

## Technical report

At our institution, in addition to the breath-hold CT (1.5 mm slice thickness), a 4DCT scan is acquired for all lung patients treated with real-time adaptive motion management, with or without fiducials. For fiducial cases, the 4DCT scan enables evaluation of the relationship between the tumor and the fiducial (if the fiducial is implanted in proximity rather than directly inside the tumor) and assessment of possible fiducial migration prior to treatment.

**Figure 1:**
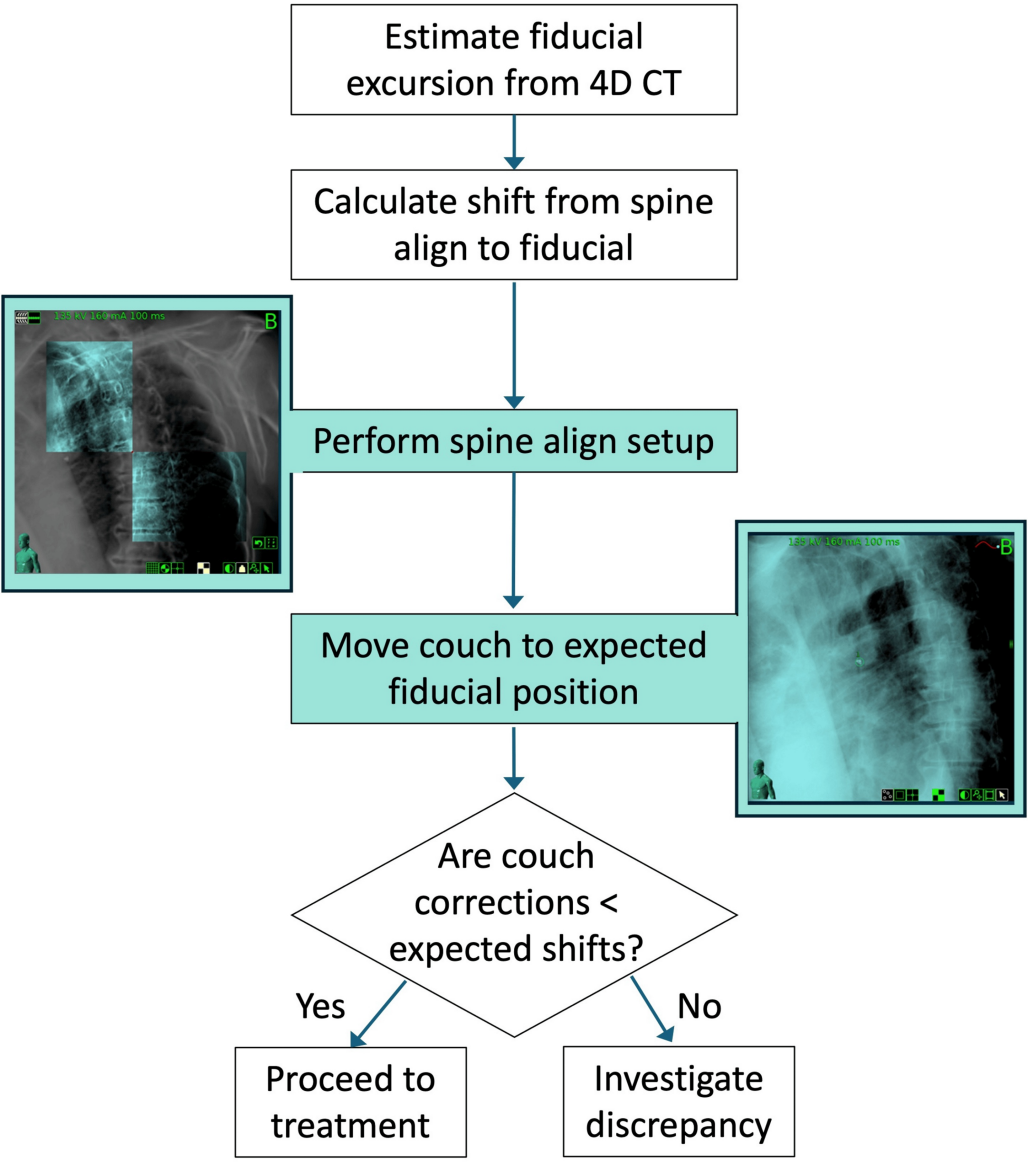
Fiducial evaluation workflow.

**Figure 2:**
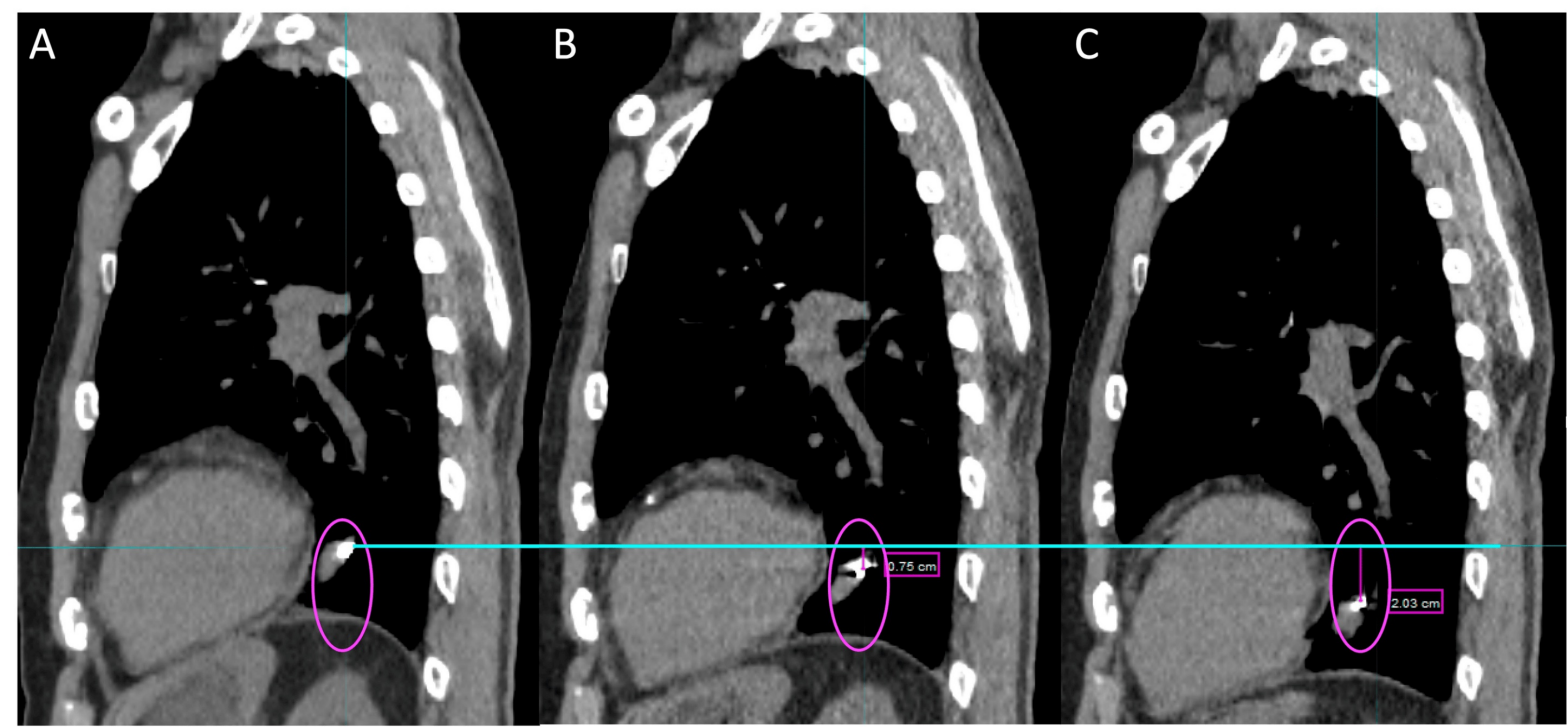
Example of sagittal images from the planning CT (A), exhale phase of the 4DCT (B), and inhale phase of the 4DCT (C), used to evaluate fiducial excursion. (A) Breath-hold exhale planning CT; (B) Exhale phase of 4DCT; (C) Inhale phase of 4DCT.

Figure 1 shows a diagram of the fiducial evaluation workflow. In MIM Software (version 7.3.2), a rigid registration is performed between the breath-hold planning CT and the 4DCT scan, focusing the alignment on the vertebral bodies at the level of the target. The fiducial excursion due to regular breathing motion is determined from the 4DCT in the superior-inferior (SI), anterior-posterior (AP), and left-right (LR) directions. As patients may exaggerate their breathing amplitude during the breath-hold scan, it is important to assess differences between the breath-hold CT and the corresponding phase in the 4DCT (Figure 2). Uncertainty in fiducial localization due to imaging artifacts in the 4DCT phases is on the order of 1-2 mm.

The range of motion of the fiducial through respiration and the expected difference in the fiducial's position from the initial spinal alignment are documented in the patient setup notes. On the Precision treatment planning system (version 3.3.1.2), a dummy lung tracking plan is created using the fiducial as the target tracking volume. In this case, after the spine alignment step, the couch is automatically moved to the expected fiducial location. To minimize the risk of accidental delivery, the dummy plan includes only one beam with 1 MU. Prior to treatment, the patient is aligned to the spine and the couch is shifted to the expected fiducial location. If an additional shift is required (residual shift), the discrepancy must be investigated. If the discrepancy cannot be resolved, a CT scan may be acquired to assess the target-fiducial relationship.

Six lung patients were included in the study. Patients were treated on the CyberKnife system with Synchrony® tracking to a dose of 50 Gy in 5 fractions. Lesion locations included right lower lobe (n=3), left lower lobe (n=1), right middle lobe (n=1), and right upper lobe (n=1). Couch shifts in three directions were recorded for each fraction (total fractions = 30; total shifts = 90) and compared to the expected shifts.

**Figure 3:**
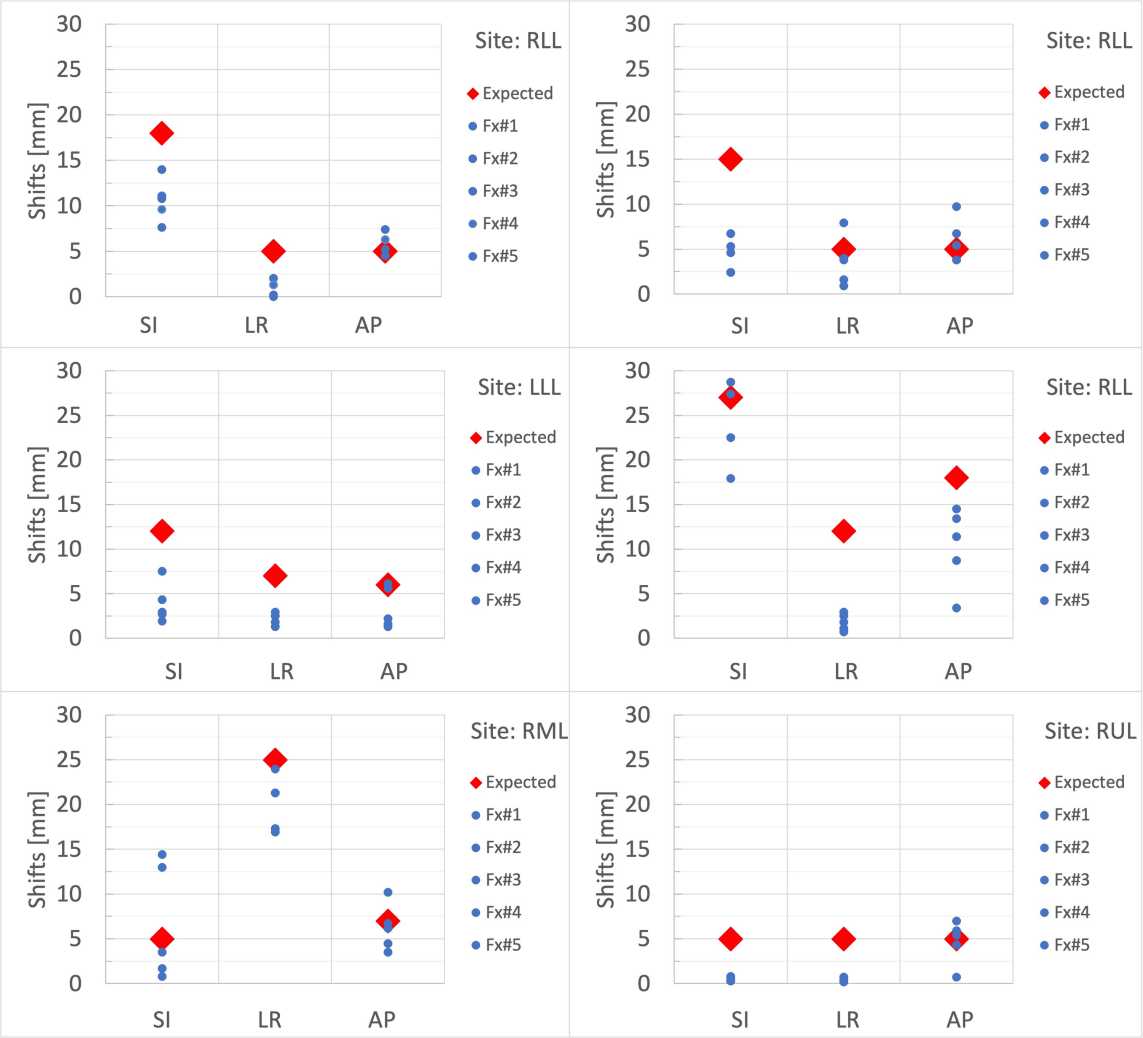
Couch shifts after the spine alignment step. The red diamonds indicate the expected shifts based on breathing motion, and the blue circles represent the actual shifts applied during each fraction. RLL: Right Lower Lobe; LLL: Left Lower Lobe; RML: Right Middle Lobe; RUL: Right Upper Lobe; Fx#: Fraction Number; SI: Superior-Inferior; LR: Left-Right; AP: Anterior-Posterior.

Figure 3 shows the expected (red diamond) and actual shifts (blue circle) for each treatment fraction. Table 1 shows the residual shifts in the three directions, the average residual shifts, and the vector magnitude. Seventeen out of 90 residual shifts (18.9%) were greater than expected, of which four (4.4%) had a >5 mm difference. Most of the residual shifts were in the AP direction (11 out of 17), while the largest residual shifts were in the SI direction (case 4 and case 5). A residual shift in two directions was observed in only one fraction. Average residual shifts over 30 fractions were 0.9 ± 2.4 mm (SI), 0.1 ± 0.5 mm (LR), and 0.6 ± 1.1 mm (AP). For each fraction, the largest average residual shift across the three directions was 3.1 mm, and the largest magnitude was 9.4 mm.

**Table 1:** Residual shifts in each direction, average residual shift, and magnitude across 30 fractions. RLL: Right Lower Lobe; LLL: Left Lower Lobe; RML: Right Middle Lobe; RUL: Right Upper Lobe; Fx#: Fraction Number; SI: Superior-Inferior; LR: Left-Right; AP: Anterior-Posterior.

Site	Fx#	SI shift (mm)	LR shift (mm)	AP shift (mm)	Average shift (mm)	Magnitude (mm)
RLL	1	0	0	0	0.0	0.00
	2	0	0	2.4	0.8	2.40
	3	0	0	0.2	0.1	0.20
	4	0	0	1.3	0.4	1.30
	5	0	0	0	0.0	0.00
RLL	1	0	2.9	4.7	2.5	5.52
	2	0	0	0	0.0	0.00
	3	0	0	1.7	0.6	1.70
	4	0	0	0	0.0	0.00
	5	0	0	0.4	0.1	0.40
LLL	1	0	0	0	0.0	0.00
	2	0	0	0	0.0	0.00
	3	0	0	0.1	0.0	0.10
	4	0	0	0	0.0	0.00
	5	0	0	0	0.0	0.00
RLL	1	0	0	0	0.0	0.00
	2	1.7	0	0	0.6	1.70
	3	0	0	0	0.0	0.00
	4	6.4	0	0	2.1	6.40
	5	0.4	0	0	0.1	0.40
RML	1	0	0	0	0.0	0.00
	2	0	0	3.2	1.1	3.20
	3	0	0	0	0.0	0.00
	4	9.4	0	0	3.1	9.40
	5	8	0	0	2.7	8.00
RUL	1	0	0	0.4	0.1	0.40
	2	0	0	0	0.0	0.00
	3	0	0	2	0.7	2.00
	4	0	0	0	0.0	0.00
	5	0	0	0.9	0.3	0.90

## Discussion

For CyberKnife patients with a single fiducial, it is not possible to validate the relationship between the target and the fiducial prior to treatment. A motion evaluation based on 4DCT combined with a spine setup plan enables a gross evaluation of fiducial migration, as well as the application of rotational correction based on spinal anatomy. Respiratory motion is not the only cause of changes in the spine-tumor relationship. The position of a lung lesion relative to the spine is influenced by day-to-day variations in breathing patterns and internal anatomy [10]. Therefore, a residual shift beyond what is expected from the respiratory motion assessment might be needed after initial alignment. As uncertainties in the spine-tumor relationship are patient-specific, it is not possible to provide an absolute threshold for the residual shift. On a case-by-case basis, the user should evaluate the magnitude of the residual shift and investigate any discrepancies as necessary. In the evaluated patients, the magnitude of the residual shift was <1 cm, and no patient had residual shifts >5 mm in two directions.

The SI direction is the largest component of motion reported for lung lesions [11], which might explain the large SI residual shifts. The application of rotational correction at the spine might contribute to residual shifts in the AP direction, particularly for lesions distant from the spine.

For fiducial-less cases, the AAPM Task Group Report 135B recommends evaluating the difference between the expected tumor location after the spine-to-lung couch shift and the actual tumor location as a way to assess tracking consistency [9]. Thresholds and action levels for this difference are not provided by TG-135B and are left for the user to determine on a case-by-case basis. Our results on fiducial patients reflect possible breathing pattern and anatomical variations from simulation to treatment and could help users make this determination.

The proposed workflow enables the detection of gross (centimeter-level) fiducial migrations. Two or more fiducials are required to detect millimeter-level migrations or deformations in the fiducial configuration. If fiducial migration is suspected, a CT scan should be acquired to evaluate internal anatomy in 3D.

## Conclusions

A spine setup plan combined with 4DCT enables the evaluation of potential fiducial migration in CyberKnife lung patients. Following the spine-to-lung couch shift, the fiducial should be located at or near the imaging center. If an additional shift is required, its magnitude should be evaluated to rule out the possibility of fiducial migration.
